# Emergency department care experiences among people who use substances: a qualitative study

**DOI:** 10.1186/s12939-023-02050-x

**Published:** 2023-12-04

**Authors:** Dana Rajab, Jamie K. Fujioka, Melanie Walker, Susan A. Bartels, Meredith MacKenzie, Eva Purkey

**Affiliations:** 1 Department of Medicine, Queen’s University, Kingston, ON Canada; 2 Department of Emergency Medicine, Queen’s University, Kingston, ON Canada; 3 Department of Family Medicine, Queen’s University, Kingston, ON Canada; 4 Department of Public Health Sciences, Queen’s University, Kingston, ON Canada; 5 Street Health Centre, Kingston Community Health Centres, Kingston, ON Canada

**Keywords:** People who use substances (PWUS), Substance use, Addictions, Emergency department (ED), Equity-deserving group (EDG)

## Abstract

**Background:**

People who use substances (PWUS) encounter significant barriers to accessing care for their complex health needs. As a result, emergency departments (EDs) often become the first point of healthcare access for many PWUS and are a crucial setting for the study of health inequities. This study aimed to understand the ED healthcare experiences of PWUS with the intent of informing ways of improving the delivery of equitable care.

**Methods:**

This qualitative study was part of a larger cross-sectional, mixed-methods study that examined ED experiences among diverse underserved and equity-deserving groups (EDGs) within Kingston, Ontario, Canada. Participants shared and self-interpreted a story about a memorable ED or UCC visit within the preceding 24 months. Data from participants who self-identified as having substance use experiences was analyzed through inductive thematic analysis.

**Results:**

Of the 1973 unique participants who completed the survey, 246 participants self-identified as PWUS and were included in the analysis. Most participants were < 45 years of age (61%), male (53%), and white/European (57%). 45% identified as a person with a disability and 39% frequently struggled to make ends meet. Themes were determined at the patient, provider, and system levels. Patient: *history of substance use* and experience of *intersectionality* negatively influenced participants’ anticipation and perception of care. Provider: negative experiences were linked to *assumption making*, *feelings of stigma and discrimination*, and *negative perceptions of provider care*. Whereas positive experiences were linked to *positive perceptions of provider care*. System: *timeliness of care* and the perception of *inadequate mental health resources* negatively impacted participants’ care experience. Overall, these themes shaped participants’ trust of ED staff, their desire to seek care, and their perception of the care quality received.

**Conclusions:**

PWUS face significant challenges when seeking care in the ED. Given that EDs are a main site of healthcare utilization, there is an urgent need to better support staff in the ED to improve care experiences among PWUS. Based on the findings, three recommendations are proposed: (1) Integration of an equity-oriented approach into the ED, (2) Widespread training on substance use, and (3) Investment in expert resources and services to support PWUS.

## Background

People who use substances (PWUS) are an equity-deserving group (EDG) that represent a significant proportion of emergency department (ED) visits [1–4]. In North America, PWUS account for approximately 1 in 11 ED encounters [4]. The COVID-19 pandemic has further exacerbated this prevalence with over 80,000 hospitalizations attributed to substance use in 2020, an increase of 4000 from 2019 [5]. PWUS are also at a higher risk of experiencing other complex social challenges including vulnerable housing and mental health challenges [6, 7].

The ED is a critical point of contact in the healthcare system for PWUS. PWUS often access the ED for health reasons other than substance use including acute and chronic illness and injuries [8]. Unfortunately, there is evidence that when seeking care in the ED, PWUS still view the hospital as a “risk environment” or “last resort”, reserved for emergencies or acute situations due to their desire to avoid negative encounters with the healthcare system [9, 10].

Qualitative studies have explored the healthcare experiences of PWUS from both a health care provider (HCP) and patient perspective, although the latter is limited. From a HCP perspective, studies noted overlapping positive and negative experiences when giving care. On one hand, some providers felt they employed empathetic care and understood the nuances involved in treating PWUS [11–18]. On the other hand, many felt ill-equipped and uncertain about how to best serve PWUS, which were noted to contribute to patient experiences of stigma and inaccessibility [11–18]. These findings highlight the importance of better understanding how care is provided to PWUS and the meaning of “appropriate and equitable care” for this patient population. From a patient perspective, care experiences of PWUS have been explored in various healthcare settings [18–23] however, very little is known about the ED context [24–27]. In non-ED healthcare settings, PWUS attributed their negative care experiences to accessibility barriers, discrimination and stigmatization, interpersonal violence and aggression, poor communication, inadequate pain management, and lack of patient centered supports [18–23]. The limited research describing ED care of PWUS noted negative experiences including breaches of trust, addictions related stigma, unsympathetic care, and hesitancy to seek future ED care [24–27]. Although the care experience of PWUS seems to be influenced by both the healthcare system and HCPs, their perception of the patient-provider relationship appeared to be a very prominent aspect of how PWUS interpreted their care experience.

The ED is a critical setting for the study of health inequities faced by PWUS because of the role it plays as the first point of healthcare access for many PWUS. Several studies established useful frameworks that offer guidance for EDs in addressing health inequities directly in the ED environment [28–30]. Such examples are the Equity Oriented Healthcare (EOHC) and Trauma and Violence Informed Care (TVIC) frameworks [28–30]. To best apply these frameworks however, it is first and foremost important to learn about the inequities experienced by this population. This study aimed to understand the care experiences of PWUS, described in their own voices, with the goal of moving towards improving their care experiences in the ED.

## Methods

### Study design

This qualitative study is part of a larger participatory, mixed-methods, cross-sectional study that examined ED care experiences among diverse EDGs in Kingston, Ontario, Canada. This study used a ‘sensemaking’ methodology with Spryng.io software. Spryng.io is a digital narrative capture tool that collects typed or audio-recorded micronarratives via handheld tablets. Sensemaking is a process by which a large number of participants self-interpret their micronarratives to help generate meaning from their own and their community’s experience [31].

### Study setting and participants

Participants were recruited via convenience sampling at a single urban ED and a single urban Urgent Care Centre (UCC) in the Kingston Health Sciences Centre (KHSC), as well as through community organizations. KHSC is a large acute and specialty care referral centre that serves a catchment of 500,000 people from surrounding urban and rural communities within southeastern Ontario, Canada. The ED and UCC at KHSC have annual patient volumes of 57,648 and 37,708, respectively.

Between June to August 2021, trained research assistants (RAs) invited patients or individuals accompanying patients presenting to the ED or UCC to participate in the study. Study inclusion criteria included any medically stable patient, aged 16 and older, with adequate English fluency registering in the ED or UCC during study hours, from 9am to 9pm Monday to Friday. Patients who were under the age of 16, were not medically stable, were aggressive towards staff or who did not have the capacity to provide informed consent were not approached to participate. To capture the experiences of those who were not actively seeking care in the ED or UCC during the study period, RAs also recruited eligible participants at community partner organizations such as the Kingston Street Health Centre (a community health centre with a focus on providing substance use and addiction services), Home Based Housing (local shelter services), St. Vincent de Paul (an organization providing food and material support for low-income people), the Kingston Youth Shelter, and the Integrated Care Hub (a location providing low barrier shelter and substance use services for people actively using substances).

### Data collection

Participants were prompted to share and self-interpret a micronarrative about their most memorable previous ED or UCC visit within the preceding 24 months. Participants responded to one of the following open-ended prompting questions: (1) *Share an example of how visiting the emergency room helped or harmed you or someone you were at the hospital with?* (2) *Tell a story about the best or worst experience you or someone you were with had in the emergency room?* or (3) *Give an example of an experience that went very well or very badly for you or someone you were with at the emergency room*. Multiple-choice questions at the end of the survey collected demographic information, allowing patients to self-identify as a member of up to three EDGs, and asked questions that contextualized the shared story (e.g., how often do the events in the story happen, who was the story about, was the story related to substance use, etc.). Each participant was assigned a unique identifier to prevent them from participating more than once.

### Data analysis

For the purpose of this analysis, micronarrative data from participants who self-identified as having substance use experiences were reviewed, analyzed, and coded using inductive thematic analysis by two independent researchers (DR and JK) [31]. Inductive thematic analysis is a phenomenological approach wherein emergent themes or patterns are generated from the raw data [31]. NVIVO 12 software was used to establish thematic nodes summarizing ED care experiences which were then collated into higher order subthemes and themes individually by each researcher. Coding, subthemes and themes were then compared, discussed and reviewed. Any conflicting views were resolved by the lead investigator (EP). Following analysis, identified themes and supporting quotes from participants were presented in a focus group format to service providers from community organizations and PWUS. This form of triangulation enhanced study credibility by eliciting feedback about the themes generated from PWUS and service providers caring for PWUS [32].

### Ethical considerations

This study was designed in collaboration with members of EDGs and community-based organizations that serve PWUS. Informed consent was recorded digitally from participants on the tablet prior to data collection. No identifying information was collected to maintain participant anonymity. Participants were offered a $5 coffee gift card as a token of appreciation for their time. The study protocol was approved by the Queen’s University Health Sciences and Affiliated Teaching Hospitals Research Ethics Board (#6,029,400).

## Results

### Study participants

Of the 1973 unique participants who completed the survey, a total of 246 participants self-identified as PWUS and were included in the study. Their micronarratives were largely collected from local sites including the ED (33%), UCC (17%), and community sites (57%). Most participants were < 45 years of age (61%), male (53%), and white/European (57%). Further, 45% of participants identified as a person with a disability and 39% frequently struggled to make ends meet.

### ED visit characteristics

For a detailed description of the ED visit characteristics of the study participants, refer to Table 1 .

**Table 1:** Participant characteristics among those who identify as PWUS compared with those who do not identify as part of an equity-deserving group.

Variable	Identifies as a Person Who Uses Substances (PWUS) (% of N = 246)	Does Not Identify as Equity-Deserving (% of N = 949)	Total Participants (% of N = 1195)	P-value*
*Gender Identity*				0.0141
Female	108 (44%)	517 (54%)	625 (52%)	
Male	131 (53%)	411 (43%)	542 (45%)	
Non-binary	1 (0%)	5 (1%)	6 (1%)	
Missing data	6 (2%)	16 (2%)	22 (2%)	
*Age*				<0.0001
< 18	6 (2%)	93 (10%)	99 (8%)	
18–25	31 (13%)	93 (10%)	124 (10%)	
26–45	92 (37%)	167 (18%)	259 (22%)	
46–65	49 (20%)	166 (17%)	215 (18%)	
> 65	8 (3%)	153 (16%)	161 (13%)	
Missing data	60 (24%)	277 (29%)	337 (28%)	
*If the patient is a person with a disability, which relates most to the story (type of disability)?*				<0.0001
Hearing loss/deafness	2 (1%)	11 (1%)	13 (1%)	
Low vision/blindness	0 (0%)	1 (0%)	1 (0%)	
Intellectual Disability	7 (3%)	3 (0%)	10 (1%)	
Physical Disability	24 (10%)	16 (2%)	40 (3%)	
Mental Health Disability	67 (27%)	14 (1%)	81 (7%)	
Other	10 (4%)	10 (1%)	20 (2%)	
Patient is not a person with a disability	63 (26%)	571 (60%)	634 (53%)	
Missing data	73 (30%)	323 (34%)	396 (33%)	
*What is patient’s sexual orientation?*				<0.0001
Asexual	0 (0%)	3 (0%)	3 (0%)	
Bisexual	36 (15%)	27 (3%)	63 (5%)	
Gay/Lesbian	4 (2%)	11 (1%)	15 (1%)	
Pansexual	8 (3%)	9 (1%)	17 (1%)	
Straight	184 (75%)	830 (87%)	1014 (85%)	
Questioning/unsure	1 (0%)	2 (0%)	3 (0%)	
Sexual orientation not on this list	0 (0%)	2 (0%)	2 (0%)	
Missing data	13 (5%)	65 (7%)	78 (7%)	
*Gender diverse*				<0.0001
Yes	11 (4%)	4 (0%)	15 (1%)	
No	223 (91%)	892 (94%)	1115 (93%)	
Missing data	12 (5%)	53 (6%)	65 (5%)	
*Frequency With Which Patient Struggles to Make Ends Meet*				<0.0001
Never	40 (16%)	524 (55%)	564 (47%)	
Rarely	24 (10%)	155 (16%)	179 (15%)	
Sometimes	48 (20%)	131 (14%)	179 (15%)	
Often	32 (13%)	34 (4%)	66 (6%)	
All the time	95 (39%)	32 (3%)	127 (11%)	
Missing data	7 (3%)	73 (8%)	80 (7%)	
*Where did the shared story take place?*				< 0.0001
Emergency Department	186 (76%)	545 (57%)	731 (61%)	
Urgent Care Centre	60 (24%)	404 (43%)	464 (39%)	
*Was the story a first-hand or second-hand experience?*				< 0.0001
First-hand personal experience	222 (90%)	771 (81%)	993 (83%)	
Second-hand experience	20 (8%)	171 (18%)	191 (16%)	
Other/Missing data	4 (2%)	7 (1%)	11 (1%)	
*How long ago was the shared ED visit?*				0.8845
0–6 months	94 (38%)	316 (33%)	410 (34%)	
7–12 months	28 (11%)	104 (11%)	132 (11%)	
13–18 months	17 (7%)	77 (8%)	94 (8%)	
19–24 months	33 (13%)	123 (13%)	156 (13%)	
More than 24 months	11 (4%)	42 (4%)	53 (4%)	
Not sure/Missing data	63 (26%)	287 (30%)	350 (29%)	
*How often do you think this situation occurs?*				0.0006
It happens all the time	100 (41%)	236 (25%)	336 (28%)	
It is somewhat typical	63 (26%)	267 (28%)	330 (28%)	
It happens from time to time	35 (14%)	159 (17%)	194 (16%)	
It is very rare	17 (7%)	91 (10%)	108 (9%)	
Missing Data	31 (13%)	196 (21%)	227 (19%)	
*How did your personal situation, identity, and culture impact the care experience?*				<0.0001
In a very bad way	56 (23%)	4 (0%)	60 (5%)	
In a bad way	41 (17%)	18 (2%)	59 (5%)	
It did not impact care	108 (44%)	715 (75%)	823 (69%)	
In a good way	14 (6%)	50 (5%)	64 (5%)	
In a very good way	7 (3%)	29 (3%)	36 (3%)	
Missing data	20 (8%)	133 (14%)	153 (13%)	
*Feelings About the ED Experience*				<0.0001
Positive	75 (30%)	550 (58%)	625 (52%)	
Negative	126 (51%)	242 (26%)	368 (31%)	
Mixed positive / negative	32 (13%)	79 (8%)	111 (9%)	
Missing data	13 (5%)	78 (8%)	91 (8%)	
*Shared Experience About Lack of Respect and/or Judgement*				<0.0001
No	123 (50%)	787 (83%)	910 (76%)	
Yes	98 (40%)	85(9%)	183 (15%)	
Missing data	25 (10%)	77 (8%)	102 (9%)	
*Number of EDG groups indicated* **Substance use, vulnerably housed, Indigenous, 2SLGBTQ+, ethnic minority, experiencing sexual assault/violence, mental health, disabilities				<0.0001
0	0 (0%)	949 (100%)	949 (49%)	
1	38 (15%)	0 (0%)	503 (26%)	
2	58 (24%)	0 (0%)	223 (11%)	
3	150 (61%)	0 (0%)	268 (14%)	

*Chi-squared tests were used and did not include missing data / “not sure/prefer not to say”

### Thematic analysis

Emerging themes were determined at the patient, provider, and health system levels. Patient-level themes consisted of *history of substance use* and the experience of *intersectionality*. Provider-level considerations were prominent with themes including *assumptions*, feelings of *stigma and discrimination*, and *perceptions of provider care*. System-level considerations predominantly focused on themes including *timeliness of care* and the perception of *inadequate mental health resources.* Although these themes were presented distinctly, they often overlapped to influence participants’ ED experiences. Each emerging theme was presented to a focus group comprised of PWUS and service providers from community organizations as a method of triangulation based on their experiences. None of our generated themes were modified following these focus groups.

## Patient-level considerations

### History of substance use

Several participants were aware of being labeled as “substance users” in their medical files whether they actively used substances at the time of the visit, and noted the impact this had on their care, and their anticipation of care before even entering the ED. They described feelings of frustration, shame, anxiety, and nervousness about how they would be treated based on previous experiences. As one woman shared: *“They pull up your form and see you’re an ex-addict so you tend to be treated with judgment” (Female, 46-65yrs).* This finding extended to participants who no longer used substances but still faced feelings of worry solely for having a documented history of substance use.

*“They just assumed that she was there for pain medication as she is marked as an addict when she has had sobriety for many years… Her experience was horrible. And it seems that every time she needs to go to the hospital, she is treated like that because of her past” (Male, 26-45yrs).*

### Intersectionality

Alongside their experience with substance use, some participants that identified with other EDGs felt the ED did not adequately meet the needs of their intersectional identities. This was articulated prominently when participants concurrently experienced substance use, housing, and/or mental health challenges.

One vulnerably housed man shared the following after being treated for an overdose:

*“After I was sick, um, they kind of just kicked me out right away. They didn’t really give me a chance to recover. So, then I was homeless at the time. So I was forced to be on the street, and I was sick, and I had nowhere to go” (Male, 18-25yrs).*

## Provider-level considerations

### Assumptions

ED staff were felt to make assumptions about participants’ substance use status, drug seeking intentions, legitimacy of their health presentation, and method of drug use. They felt that this led to staff focusing on concerns that did not align with patient expectations and was perceived as delayed/and or inadequate care. Downstream effects of this were negative impacts on the patient-provider relationship and fostering feelings of stigma and discrimination.

*“I have to say from that experience of being with them realizing that I had a legitimate problem, and I was in actual pain that I was drug-seeking for a reason because I was losing my mind, with the pain… I am on methadone, low dose. Um, but when they see methadone they judge you. It is always you abuse drugs, that you use needles. And that is not always the case. I use methadone now because I have peripheral neuropathy from all of the chemo I took.” (Male,45-54yrs).*

### Stigma and discrimination

Stories of participants facing feelings of stigma and discrimination were prominently expressed. They shared the perception that once staff knew or suspected substance use their attitudes and treatment of participants quickly changed.

*“The last time I went to [hospital] it was because I had a drug problem. And I found that the staff treatment was once they found out that I was on drugs they treated me like garbage. And I wish that would change.” (Female, 26-45yrs).*

Negative treatment towards participants included experiences of rough care, negative comments, and care that was felt to lack respect and empathy. One woman shared her story of staff mistreatment of her daughter when she visited the ED with an abscess:

*“They weren’t very polite either to her or to me. And I told them that you know, she may be a drug addict to them, but she is my daughter. And she needs to be treated with respect. And the doctor came in and looked at her abscess and said well you know we are going to have to lance it. And my daughter asked if he would freeze it first. And he said well what the hell is the difference. You know a poke or a cut. And as he said that he just took the scalpel and he cut her arm open. And of course, my daughter is crying because it hurt. And, um, he was being very rough with her.” (Female, 26-45yrs).*

The feeling of a lack of non-judgmental care led to participants feeling uncomfortable disclosing their use of substances and in some cases, choosing to terminate the care visit prematurely.

*“I went to [hospital] because I was having a minor heart attack as I thought. Um, I was sitting in the waiting room and was told that if I was not on methadone I would not be there. That I was just a junkie and that didn’t deserve to have healthcare. I went into the room, and it was the same thing, but I had a towel thrown at me and I was told that I was not worth the bed that I was in. And I felt like crap, and so I went home, and my doctor told me I was going to have a heart attack.” (Female, 26-45yrs).*

### Perceptions of provider care

Participants shared both positive and negative experiences pertaining to how they felt that ED staff cared for them. Positive perspectives were related to ED staff being welcoming and professional, providing comfort and emotional safety, effectively communicating, being efficient in their care, and providing resources.

*“So, my last experience I had at [hospital] I brought somebody in for alcohol withdrawal. And in doing so they triaged her and brought her right into a bed. They gave her fluids immediately. And the attending physician and the nurses were very kind. They spent lots of time with her trying to find out if there was some way to help and get a bed at detox. They kept her there as long as they could until she was able to walk herself. They wanted to keep her there for longer so that she could get more stable. Um, and further away from alcohol their idea was obviously that would be better. The attending physician brought me aside and asked me if it was safe for her to go home. They were kind, they were thorough, um, there was no sense of judgement, no sense of rush.” (Male, 55-65yrs).*

Negative perspectives were related to the perception of rude staff, perceived patient neglect, feelings of inadequate supportive care, and lack of patient-centered care. Lack of patient-centered care was characterized by feelings of not being listened to, not receiving referrals personalized to their needs, and a lack of continuity of care. Patient neglect was often identified in stories of overdose, however, not always.

*“My mother had stage 4 lung cancer. I had to bring her to emerg at [hospital]. She was a drug user and was treated as so. She was skipped, no one wanted to give her pain meds because she was an addict. We also did have some very nice nurses and doctors but certainly mistreated lots.” (Female, 24-34yrs)*.

*My friend brought me in after I fainted and he didn’t know why. Everyone immediately rushed to me to get looked at, and after learning it was an overdose they didn’t keep it as a priority. They put me in the waiting room while I was overdosing and didn’t even check on me. I needed serious medical attention and was pushed aside. I couldn’t keep my eyes open and kept getting sick, couldn’t breathe and they just left me there. (Female,18-25yrs)*

## System-level considerations

### Timeliness of Care

Participants discussed experiences of both timely and untimely care. Time-sensitive care was described when the ED urgently addressed health concerns in what participants felt to be a *“decent amount of time”* and quickly directed participants to their next steps.

*“I accompanied my brother to the [hospital]. He was needing a prescription for Valium as he was going to Detox. While in the waiting area he had a seizure and fell to the floor. I called for help and there was an immediate response from hospital personnel who got him stabilized and onto a gurney. They were very responsive in the care and reassuring to me. once settled, He was given 5 mg of Valium” (Female, 55-64yrs).*

On the other hand, a majority of participants articulated untimely care associated with long wait times that they perceived to be linked to their identity as a PWUS. The ED environment was described as understaffed and/or overworked leading to many participants waiting with challenging health circumstances. Long-wait times were most often discussed in the context of triage and described as *“excessive”* resulting in participants feeling frustrated and *“passed over”* with some making the decision to leave the ED without receiving care.

*“It would seem that they need more staff on Mondays…because patients were trying to leave because they thought they were in the way of the health team. And that shouldn’t be a thought that they are having if their condition is life-threatening. Um, so yeah, more staff on Mondays. The experience was bad again, not their fault. All of the people who have helped me have been lovely and very, very personable. But the system that they are working in is not there to serve them” (Female, 16-24yrs).*

### Inadequate mental health resources

Participants with both substance use and mental health experiences often spoke about the perception of the lack of available and accessible mental health resources in the ED to meet their specific needs. These experiences covered descriptions of inadequate mental health programs, difficulties connecting with psychiatrists in the ED, and the challenge of choosing whether to discharge patients presenting to the ED with mental health concerns. One man shared:

*“I came 2 months ago, over mental health issues. I was seen in reasonable amount of time, although the results were less than satisfying. I was referred to multiple programs, but none of them were actually suited to my needs. Almost as if, they were “automatic” referrals and I hadn’t actually been listened to” (Male, 25-34yrs).*

## Discussion

This study sought to elicit the care experiences of PWUS in the ED to better understand their challenges in seeking care, in hopes of informing more inclusive and equitable ways to improve ED experience and access in future. It was found that the care experience of PWUS was influenced by patient, provider, and system level factors. The challenging and unique societal experiences of PWUS were prominent in our study findings, reflecting existing literature. Assumption-making, stigma, and discrimination were prevailing issues found in the ED that contributed to mistrust, perceptions of negative and poor treatment, and ultimately, patients opting to avoid or leave care. These consequences propagated the experience of inequitable and unmet healthcare needs. These findings are in line with other studies which discuss the profound and long-lasting mark left on patients by the enacted and anticipated stigma they face in healthcare settings [18, 24–27, 33, 34]. A finding that was not prominent in our study but has been described elsewhere is that the type of substance use influences the degree of stigma experienced [33]. For instance, people who inject drugs or use methadone face greater levels of stigmatization in comparison to people who use alcohol [33]. This reinforces the importance of implementing patient-centered care that treats patients based on their unique experiences and destigmatizes all substance use.

PWUS were found to truly remember their interactions with HCPs and staff in the ED. This emphasizes the power healthcare providers hold in bridging the gap between positive and negative ED experiences for the patients they serve. Negative experiences were rooted in the perception that ED staff were not authentically present to care for the patient. This included the absence of patient-centered care, perceived patient neglect, inadequate supportive care, and the perception of rude or stigmatizing staff; all of which have been found by other studies [9, 10, 19, 23, 24, 26, 27, 34]. Importantly, however, in our study and elsewhere positive interpersonal experiences arose where PWUS felt welcomed, comforted, and heard, and when they received well communicated, efficient care [9, 24, 26, 34–37]. A mixture of positive and negative experiences suggests that healthcare providers may generally hold the intention to maintain and foster positive relationships with their patients but are experiencing obstacles to providing high-quality, patient-centered care to this vulnerable population, leading to negative experiences, and this requires further study.

A potential explanation underlying negative experiences with healthcare staff is the current state of EDs. In many nations including Canada, EDs are operating under considerable pressures and limited capacities. Although the COVID-19 pandemic has undoubtedly exacerbated this issue, this is nevertheless not a new problem [38]. In such stressful and time-sensitive environments, healthcare staff are more likely to face personal and emotional stresses, burnout, and exhaustion [38, 39]. In fact, over 50% of ED physicians reported feelings of distress [38, 40]. The sequelae of physician distress and a strenuous work environment may lead to patient depersonalization, worsened interpersonal relationships with patients, decreased patient satisfaction, and ultimately reduced quality of care [38, 39]. PWUS are a group that repeatedly faces great structural challenges in obtaining appropriate care to meet their needs and so rely on EDs for primary care [41]. Consequently, they may present frequently and may be judged as an “unnecessary ED visit” in an already overworked and crowded ED environment [27, 41]. This opens the opportunity to intervene at the structural level to support ED staff in providing adequate and compassionate care to equity deserving populations, as well as creating alternate points of access for non-emergent health concerns. Furthermore, the structural stigma experienced in the ED is mirrored by the widespread stigma in society against substance use which reinforces the devaluation and de-prioritization of PWUS [42]. Therefore, interventions are also needed to de-stigmatize perceptions of PWUS. Finally, substance use and mental health services are commonly underfunded and under-resourced which perpetuates the inaccessibility of these health services among PWUS [43, 44].

### Recommendations

Based on this study’s findings and feedback from focus group discussions in the community, our proposed recommendations are three-fold:

#### Integration of equity oriented framework approaches

HCPs possess the power to address health inequities directly at the point of care. Established frameworks such as the Equity Oriented Healthcare Framework (EOHC) by Equipping Health Care for Equity (EQUIP) BC offer useful guidance for healthcare providers in the ED and primary care settings [28, 30, 45]. The EOHC framework consists of three key dimensions that must be contextually tailored: (1) Provision of Culturally Safe Care, (2) Trauma and Violence Informed Care (TVIC), and (3) Harm-Reduction [28, 30, 45]. Based upon this framework, the EQUIP intervention was designed with a dual focus on staff education and organizational-level integration [28, 30, 45]. From the staff level, this includes educational methods, enhancing staff’s knowledge and capacity to provide EOHC, further training on harm reduction and trauma informed care, and a focus on navigating care for PWUS [28, 30, 45]. From an organizational level, this includes shifts in funding, structures, practices, and policies [28, 30, 45]. This intervention has been tested in EDs and primary healthcare settings in Canada and shown improvements in self-reported patient outcomes, and enhanced staff comfort and confidence in providing EOHC [29, 30, 45, 46].

#### Widespread training specific to substance use

Substance use is a relatively common phenomenon with approximately 21% of Canadians (6 million) experiencing substance misuse or addiction in their lifetime [47]. Due to this high prevalence, ED health professionals will regularly come into contact with PWUS. Therefore, training specific to understanding and navigating substance use encounters, treatment, management, withdrawal, and specific resources must be implemented [48]. Particular attention must also be placed on decolonizing approaches, intersectionality, and de-stigmatization [28]. It is vital for training to be delivered widely across all clinical professionals interacting with PWUS, including trainees. Furthermore, it is pertinent to include individuals with lived experiences in developing and delivering training curricula as educators, peer support workers, and training creators [44]. Finally, research suggests that longer consistent training over time is more likely to produce meaningful effects rather than a one-time session [44, 49].

#### Investment in expert resources and services to support PWUS

Expert healthcare teams such as addiction consulting services (ACS) are comprised of physicians, nurses, social workers, and peer support workers that can work alongside ED physicians to provide comprehensive substance use related services [23]. These include substance use focused history taking, physical examinations, withdrawal management, and initiating pharmacotherapies [23]. They are also well equipped to ensure the transfer of care to primary and community providers, enhancing the continuity of care within the system for PWUS [23]. Although these services are scarcely available in North America, they have been demonstrated to successfully improve patient care [23, 50, 51].

### Strengths and limitations

This study is not without limitations. The main limitation is that our study did not explore the perspective of ED health providers. Additional research should expand on this important perspective and provide comparison to our findings. Moreover, data were collected from a convenience sample of potential participants who sought care during study hours and therefore the sample may not be representative of the Kingston community. Furthermore, it is possible that the patient characteristics and substance use challenges of our study population differ from other locations and may not be generalizable to all EDs. However, our findings are closely aligned with existing literature conducted worldwide. Finally, our study did not stratify experiences based on the type of substance use which makes it more challenging to ascertain the extent of which this may or may not influence their care experiences.

Despite its limitations, this study has many strengths. To begin, this qualitative study incorporated a large sample size of PWUS from a small city with a high prevalence of substance use. Detailed patient characteristics were collected including but not limited to socioeconomic status, mental health status, vulnerable housing status, indigeneity; all of which provided insight into the study population. By using a ‘sensemaking’ approach, participants were empowered to share whatever aspect of the ED care was most important to them. By collecting data at community partner organizations, we engaged with those who may no longer be accessing the ED due to prior negative experiences and in doing so, likely reduced selection bias. Key findings were shared with PWUS in a focus group discussion and with service providers who support PWUS to ensure that results were being interpreted appropriately, thus reducing interpretation bias.

## Conclusion

PWUS encounter numerous inequities when seeking ED care at personal, provider, and system-levels. As a result, they receive care that they perceive as inadequate, require frequent ED visits, avoid seeking care, and leave without care. Further studies need to evaluate the clinical, environmental, and structural interventions in the ED and prioritize integrating provider perceptions when tailoring interventions for improvement to advance equity for this patient population.

## Data Availability

The datasets used and/or analyzed during the current study are available from the corresponding author on reasonable request.

## List of Abbreviations

PWUS: People Who Use Substances
ED: Emergency Department
EDG: Equity Deserving Group
HCP: Health Care Provider
EOHC: Equity Oriented Healthcare
TVIC: Trauma and Violence Informed Care
UCC: Urgent Care Centre
KHSC: Kingston Health Sciences Centre
EQUIP: Equipping Health Care for Equity
ACS: Addiction Consulting Services
